# Oncogenic Role of Guanylate Binding Protein 1 in Human Prostate Cancer

**DOI:** 10.3389/fonc.2019.01494

**Published:** 2020-01-10

**Authors:** Jing Zhao, Xiangyu Li, Lan Liu, Jing Cao, Mariusz Adam Goscinski, Huijie Fan, Huixiang Li, Zhenhe Suo

**Affiliations:** ^1^Department of Oncology, The First Affiliated Hospital of Zhengzhou University, Zhengzhou, China; ^2^Department of Oncology, Zhengzhou University, The Academy of Medical Science, Zhengzhou, China; ^3^Department of Pathology, The First Affiliated Hospital of Zhengzhou University, Zhengzhou, China; ^4^Department of Pathology, The Third Affiliated Hospital of Zhengzhou University, Zhengzhou, China; ^5^Department of Surgery, The Norwegian Radium Hospital, Oslo University Hospital, Oslo, Norway; ^6^Department of Pathology, The Norwegian Radium Hospital, Oslo University Hospital, Institute of Clinical Medicine, University of Oslo, Oslo, Norway

**Keywords:** guanylate binding protein 1, prostate cancer, CRISPR/Cas9, xenotransplantation, energy pathway

## Abstract

The Guanylate binding proteins (GBPs) are a family of large GTPases and the most studied GBP family member is the guanylate binding protein 1 (GBP1). Earlier studies revealed that GBP1 expression was inflammatory cytokines-inducible, and most of the studies focused on inflammation diseases. Increasing number of cancer studies began to reveal its biological role in cancers recently, although with contradictory findings in literature. It was discovered from our earlier prostate cancer cell line models studies that when prostate cancer cells treated with either ethidium bromide or a cell cycle inhibitor flavopiridol for a long-term, the treatment-survived tumor cells experienced metabolic reprogramming toward Warburg effect pathways with greater aggressive features, and one common finding from these cells was the upregulation of GBP1. In this study, possible role of GBP1 in two independent prostate cancer lines by application of CRISR/Cas9 gene knockout (KO) technology was investigated. The GBP1 gene KO DU145 and PC3 prostate cancer cells were significantly less aggressive *in vitro*, with less proliferation, migration, wound healing, and colony formation capabilities, in addition to a significantly lower level of mitochondrial oxidative phosphorylation and glycolysis. At the same time, such GBP1 KO cells were significantly more sensitive to chemotherapeutic reagents. Xenograft experiments verified a significantly slower tumor growth of the GBP1 KO cells in nude mouse model. Furthermore, GBP1 protein expression in clinical prostate cancer sample revealed its aggressive clinical feature correlation and shorter overall survival association. Collectively, our results indicate a pro-survival or oncogenic role of GBP1 in prostate cancer.

## Introduction

The Guanylate binding proteins (GBPs) are a family of large cytokine-induced GTPases (1). To date, seven human GBPs (GBP-1 to -7) and 11 mouse GBPs (mGBP-1 to -11) have been described, and all human GBPs located within a single cluster on chromosome 1 (2, 3). The guanylate binding protein 1 (GBP1) is an important member of the large family of GTPases. The GBP1 gene locates on 1p22.2 at chromosome locationchr1:89,519,037- 89,528,917, and most of the available knowledge about GBPs is related to human GBP1. The structure of human GBP1 has been resolved and comprises two domains: an N-terminal globular domain with GTPase activity and a C-terminal α-helical domain (4, 5).

Human GBP1 was firstly regarded as an interferon gamma (IFN-γ) response factor and a large self-activating GTPase, playing an essential role in mediating the antiviral activities of IFN-γ (1, 6–8). It was later confirmed that not only IFNs, but also inflammatory cytokines could induce the expression of GBP1, and human GBP1 has been detected *in vivo* in inflamed tissues connected with various diseases such as cutaneous lupus erythematosus, psoriasis and Kaposi's sarcoma (9–11). Previous studies on antiviral effects have shown that human GBP1 acts against various RNA viruses such as vesicular stomatitis virus, encephalomyocarditis virus, influenza A virus, classical swine fever virus, and hepatitis C virus (12–16). Furthermore, GBP1 overexpression is associated with malignant features in different tumor types, such as glioblastoma (17), oral cancer (18), esophageal squamous cell cancer (19), ovarian cancer (20) and lung cancer (21). Increasing evidence indicates an important role of GBP1 in cancer cell growth, invasion/migration and metastasis (21–23). In addition, GBP1 was also observed to be associated with drug resistance and radioresistance in cancer cells (21, 24–28).

In our previous studies of prostate cancer cells, we firstly established the mitochondrial DNA depleted DU145 cell line by long-term ethidium bromide treatment and then the flavopiridol resistance DU145 cell line by long term flavopiridol treatment *in vitro* (29, 30). Both cell lines were revealed with metabolic reprogramming toward Warburg effect and cancer stem cell features. Transcriptomic analysis of the cell lines discovered significantly upregulated GBP1 expression in both cell lines, compared to the parental cells, with 6.78- and 8.78-fold changes for the ethidium bromide treated cell line and the flavopiridol treated cell line, respectively, strongly indicating a oncogenic role of GBP1 in prostate cancer cells. Therefore, we decided to study the GBP1 protein expression and its clinicopathological correlation in a series of prostate cancer samples, and then further explore its molecular biological consequences by performing GBP1 gene knockout (KO) in prostate cancer cell lines DU145 and PC3.

## Materials and Methods

### Cell Lines and Culture Conditions

The human prostate cancer cell lines DU145 and PC3 were obtained were obtained from ATCC (American Type culture collection, USA) and maintained in our laboratory for the study. The cells were routinely cultured in phenol red-free RPMI-1640 medium (Gibco, 11835-063, USA) supplemented with 10% fetal bovine serum (Gibco, 16000-044, USA), 100 U/ml penicillin and 100 μg/ml streptomycin (Gibco, 15140-122, USA at 37°C with 5% CO_2_.

### Generating Stable GBP1 Gene KO Cell Lines

To establish GBP1 gene KO stable cell lines, we used the CRISPR/Cas9 technology. Single guided RNA (sgRNA) sequence was generated by CRISPR design tool (http://crispr.mit.edu) and the sgRNA targeted DNA sequence was then cloned into a lentiCRISPR/Cas9 v2 plasmid. The sgRNA targeted sequence in the human GBP1 exon 2 is shown as below: TTACACAGCCTATGGTGG. When grew in 50–60% confluent, the cells were transfected with the CRISPR/Cas9 GBP1 plasmid together with Lipofection 2000, followed by 3 days puromycin selection. The cells were then harvested, diluted to single cell suspension in a density of 1 cell/100 μl, and redistributed in 96-well plate with 100 μl/well cell suspension in culture for 2 weeks for cell cloning. Monoclonal cells were obtained after two rounds of such cloning, and DNA isolated from such cells was subjected for mutation analysis.

### Mutation Analysis

The identification of GBP1 mutation was performed with PCR product sequencing. DNA was extracted from ~1 × 10^7^ cells using Genomic DNA Mini Kit (Invitrogen). The primers were: forward 5′-TACTTTGACAATACTTCCATAAC-3′ and reverse 5′-CCCCTAGAACAGCGTGA-3′, with a product length of 529 bp. The PCR reagents consisted of 12.5 μl Taq Master Mix (CWBIO, CW0682, China), 1 μl of each primer and 2 μl of DNA template. The PCR program was performed as below: initial denaturation at 94°C for 2 min, then 40 cycles of 94°C/30 s, 55°C/30 s and 72°C/30 s, plus a final 72°C extension for 2 min. The PCR products were subjected to sequencing by Sangon Biotech (Shanghai, China).

### Western Blotting Analysis

Whole cell extracts were prepared using RIPA buffer supplemented with1x Halt™ Protease/Phosphatase Inhibitor Cocktail (Thermo Scientific, USA). Protein lysates were resolved by SDS-PAGE, transferred to a PVDF membrane and incubated with primary antibodies. Antibodies against GAPDH (R&D, AF5718, USA, 1:1000), ACTIN (R&D, AF4000, USA, 1:1000), GBP1 (Abcam, ab131255, US, 1:500), and EGFR (Abcam, ab52894, US, 1:500) were applied in this study. After blocking with 5% non-fat dry milk in Tris-buffered saline with Tween-20 (TBST) for 2 h at room temperature, the immunoreactive proteins were visualized by ECL Plus kit (Thermo Scientific, USA).The Western blotting experiments were repeated at least three times and the protein bands were quantified by the Image Lab 2.0 Software.

### Assessment of Cell Proliferation Kinetics

Cells were seeded into 96 well plates at a density of 3,000 cells/well for DU145, DU145 GBP1 KO, PC3, and PC3 GBP1 KO, and cultivated for 12 h for cell attachment. The cells were then placed into an IncuCyte ZOOM for real-time phase contrast imaging and cell growth data generation. For each experiment, 3 parallel wells were prepared for each cell type, and each experiment was repeated for at least three times.

### Cell Cycle Analysis

For analysis of cell cycle phase distribution, cells in logarithmic phase were dissociated by using trypsin, and 2 × 10^6^ cells were carefully collected. The cells were fixed in pre-cooled 70% ethanol at 4°C overnight, treated with 2 μg/ml RNaseA and stained with 10 ug/ml PI, washed and prepared into single cell suspensions before DNA contents were measured with a flow cytometer (Beckman Cyto FLEXFCM USA). The Software FlowJo Version 7.6 was used for further analyses of the data.

### Transwell and Wound Healing Assays

For transwell assay, 1 × 10^5^ cells were prepared in serum- free medium and added to the upper chambers of the 24-well plate (8 μm, Transwell, Corning, USA). The plate wells were filled with 600 μl medium containing 10% of FBS. After 24 h of incubation at 37°C in a 5% CO_2_ atmosphere, the inserted chambers were fixed with methanol and stained by 0.1% of crystal violet in methanol. The unpenetrated cells were carefully removed with cotton swabs and the polycarbonate membranes were dried under room temperature. Under the microscope, cell motility was evaluated by counting the migrated cells in the lower surface of the filter. For wound healing assay, 5 × 10^5^ cells were seeded into 6-well plates and maintained until nearly 90% confluence, then wounds were made on the single cell layers with sterilized 10 μl pipette tips. After washed twice with PBS, 2 ml serum-free medium was added to the plate. The wound width was measured after 0, 12, 24, 36, and 48 h. The average scratch width was defined by using Image J software and the obtained data was used for further calculation of the relative healing speed [decreased wound width (μm)/each time point]. Each experiment was performed for at least three independent times.

### Energy Pathway Analysis

Cell mitochondrial energy metabolism including OCR and ECAR was performed using a SeahorseXFe96 analyzer (Seahorse Bioscience, USA). Cells were seeded at a density of 3 × 10^4^ cells/well in XFe96 cell culture microplate in 200 μL of growth medium and incubated at 37°C in 5% CO_2_ for 12 h and the calibrator plate was hydrated in a CO_2_-free incubator overnight. Prior to the assay, growth medium was changed to assay medium (unbuffered DMEM, 10 mM glucose, 2 mM sodium pyruvate and 2 mM glutamine.) and cells were incubated in a CO_2_-free incubator at 37°C for 1 h to allow for temperature and pH equilibration. The analyzer plotted the values of OCR and the corresponding ECAR followed by sequentially adding to each well 20 μl of oligomycin, 22.5 μl of FCCP, and 25 ul of rotenone and antimycin A mixture, to reach working concentrations of 1, 1, 0.5, and 0.5 μM, respectively. The data was analyzed with the software Wave (version 2.2.0, Seahorse Bioscience) for further visualization.

### Chemosensitivity Assay

The chemosensitivity was measured with both IncuCyte ZOOM and Colony formation assay. For IncuCyte ZOOM application, the cells were seeded at 3000/well in 96-well cell culture plates cultivated for 12 h before the cells were attached. For concentration optimization, different concentrations of docetaxel (Selleck, Catalog No. S1148) and paclitaxel (Selleck, Catalog No. S1150) were added to the culture medium of cells in preliminary experiments. Then DU145 control and experimental cells were exposed continually to docetaxel (0 nM-control, 0.1, 0.25, 0.5 nM) and paclitaxel (0 nM-control, 0.5, 1.0, 2.0 nM) and cultivated in the incubator of IncuCyte ZOOM for 96 h, while the control and experimental PC3 cells were exposed continually to docetaxel (0 nM-control, 0.5, 1.0, 2.0 nM) and paclitaxel (0 nM-control, 1.0, 2.0, 4.0 nM) and cultivated for 144 h. Attention was paid to have the same concentration of DMSO solvent in both control and experimented cells.

For colony formation assay, 1 × 10^3^ cells were seeded into 60 mm dish and cultivated overnight before the cells attached. A series of various concentration of drugs were pretested to optimize the dose application. Then different concentrations (0, 0.1, 0.25, and 0.5 nM) of docetaxel and (0, 0.5, 1.0, and 2.0 nM) paclitaxel were added to the media of DU145 and DU145 GBP1 KO for 12 days culturing before colony formation ability was evaluated. For PC3 and PC3 GBP1 KO cells the concentration had been set as docetaxel (0, 0.2, 0.4, and 0.8 nM), and paclitaxel (0, 1.0, 2.0, and 3.0 nM). Then cells were fixed with 4% paraformaldehyde (PFA) and visualized by staining with 0.1% (w/v) crystal violet in methanol. The dishes were then washed and dried before the colonies were counted in a G: BOX F3 multifunction imaging system with related software (Syngene, UK). For each experiment, 3 parallel wells were prepared for each cell type, and each experiment was repeated for at least three times.

### GBP1 Blockade Experiment With NSC756093

Briefly, the cells were cultured in RPMI 1640 medium (Gibco, 11835-063, USA) supplemented with 100 U/ml of penicillin and 100 μg/ ml streptomycin with 10% fetal bovine serum (FBS, Gibco, 16000-044, USA) at 37°C with 5% CO2. NSC756093 (SML1310, Sigma, USA), a chemical that was known as GBP1 inhibitor, was added to a final concentration of 4 μM for further experiments (31). Dose optimization of NSC756093 (Sigma) ranging from 1 to 20 μM in these cell lines was performed, and it was discovered that 4 hM was optimal for both DU145 and PC3 control cells, and therefore such concentration was applied in the GBP1 KO cells with DU145 and PC3 as controls. The IncuCyte ZOOM system was applied for NSC756093 growth influence analyses and colony formation assay and transwell assay were performed to study its inhibition effect in this study.

### Tumor Xenograft Model

BALB/c nude mice were purchased from Beijing Vital River Laboratory Animal Technology Company Limited (Beijing China) and housed in a specific pathogen–free facility. All animal protocols were performed in accordance with the guidelines of the Institutional Animal Care and Use Committee of The First Affiliated Hospital of Zhengzhou University. For subcutaneous injections, 1 × 10^7^ cells were resuspended with 100 μl of 1 × PBS and injected into right flank of BALB/nude mice (male, 4 weeks of age). Tumor volume and body weight (g) was measured every 3 days. About 4 weeks later, all the animals were sacrificed, and tumor tissues were harvested, photographed, measured. Tumor volume was calculated according to the following formula: Volume (mm^3^) = [width^2^ (mm^2^) × length (mm)]/2. Harvested tumor tissues were placed in liquid nitrogen and then frozen at −80°C or fixed in 10% buffered formalin, embedded in paraffin, sectioned and stained.

### Clinical Samples

Prostate cancer patients were eligible if they underwent transurethral resection of prostate without preoperative chemotherapy or radiotherapy during the period 2005–2012 at The First Affiliated Hospital of Zhengzhou University, Henan, P. R. China. In total, 105 patients met the eligibility criteria. The study was approved by the Ethics Committee of The First Affiliated Hospital of Zhengzhou University. All the patients involved provided written informed consent. The patients' clinical information and tumor parameters are listed in **Table 2**. The ages of the patients at diagnosis ranged from 51 to 92 years, with a median of 70 years. Tumors were classified in terms of the International Union against Cancer (UICC) 2014 standard. The Gleason score was reassigned based on the current grading recommendation provided by the International Society of Urological Pathology, there were 33 low-grade, 43 moderate-grade, and 29 high-grade tumors. Overall survival was calculated from the date of diagnosis to the date of death. The last date of follow-up was August 31, 2017. Patient follow-up information was available for a minimum period of 5 years. All the tumors were histologically classified based on the Gleason system. Two pathologists at the Department of Pathology of The First Affiliated Hospital of Zhengzhou University reviewed the type and grade of histology of the specimens.

### Immunocytochemistry (ICC) and Immunohistochemical (IHC) Staining

Cytoblocks were prepared for ICC. Cells from each cell line in 80% confluence were removed from the culture dish with trypsin and EDTA (Sigma), washed and centrifuged at 2,000 rpm for 5 min. After the supernatant were discarded, 4 drops of plasma and 2 drops of thrombin were added to the sedimentation before carefully mixed for 1 min. Then 4% buffered formalin was added to the mixture. The coagulated mass was then wrapped in a linen paper for further conventional paraffin block making process. Four micrometer sections made from these blocks were used for immunocytochemistry.

ICC and IHC were applied on the formalin-fixed, paraffin-embedded sections using the DakoEnVision™ Flex+ System (K8012; Dako, Glostrup, Denmark) and the Dako Auto stainer. After deparaffinization, rehydrating, antigen retrieval, tissue sections were treated with peroxidase blocking for 5 min, followed by incubation at 4°C overnight with rabbit polyclonal antibody against human GBP1 (1:100, ab131255, Abcam, Cambridge, UK), EGFR(1:100, ab52894, Abcam, Cambridge, UK).Then sections were washed with DAKO wash buffer for three times and incubated with rabbit linker for 15 min and EnVision™ Flex/HRP (horse radish peroxydase) enzymes for 30 min at room temperature. The staining was visualized using 3'3-diaminobenzidine tetrahydrochloride (DAB) and counterstained by hematoxylin. Known GBP1 positive human cervical cancer tissue was used as positive control, and none-immune rabbit IgG serum in exactly the same antibody concentration was used as negative control in this study. All controls showed consistent satisfactory results during the study.

### IHC Scoring System

The intensity of the IHC staining was scaled by 0 to 3 and the percentage of positive cells was scaled by 0 to 3 (Table 1). The sum of intensity score and percentage score was seen as total score, which ranged from 0 to 6. The slide was regarded as GBP1 negative, low expression and high expression when the total score is 0, 1 to 4, and 5 to 6, respectively. Examination of immunostaining was performed by two pathologists from the Department of Pathology of the First Affiliated Hospital of Zhengzhou University with consensus.

**Table 1:** The criteria of the Allred scoring system used for evaluating GBP1 expression in esophageal squamous cell carcinoma cells in our study.

1. The criteria of the intensity scoring system				
Intensity score	0	1	2	3
Staining intensity	Negative	Weak	Moderate	Strikingly positive
2. The criteria of percentage scoring system				
Percentage score	0	1	2	3
Stained cells (%)	0	<25	25–50	>50
3. Total score^a^				
	0	1–4	5–6	
	(–)	(+)	(++)	

a *The total score was obtained by adding the percentage score to the intensity score. It ranges from 0 to 6*.

### Statistical Analyses

All statistical analyses were performed with the SPSS 21.0 software program (SPSS Inc., Chicago, DE, USA) and GraphPad prism 6.0. The associations between the expression of GBP1 and categorical variables were assessed by Chi-square tests (Pearson and linear-by-linear as appropriate). Survival curves were plotted through the Kaplan–Meier method, and groups were compared with log-rank tests. The data gathered *in vitro* experiments were expressed as mean ± SD from at least three independent experiments. The one-way ANOVA (more than two groups) and *t*-test (two groups) were applied for the comparison of individual variables in the right way to analyze the difference between each subgroup. *P* < 0.05 was considered statistically significant.

## Results

### The GBP1 Gene KO Cell Line Identification

**Figure 1:**
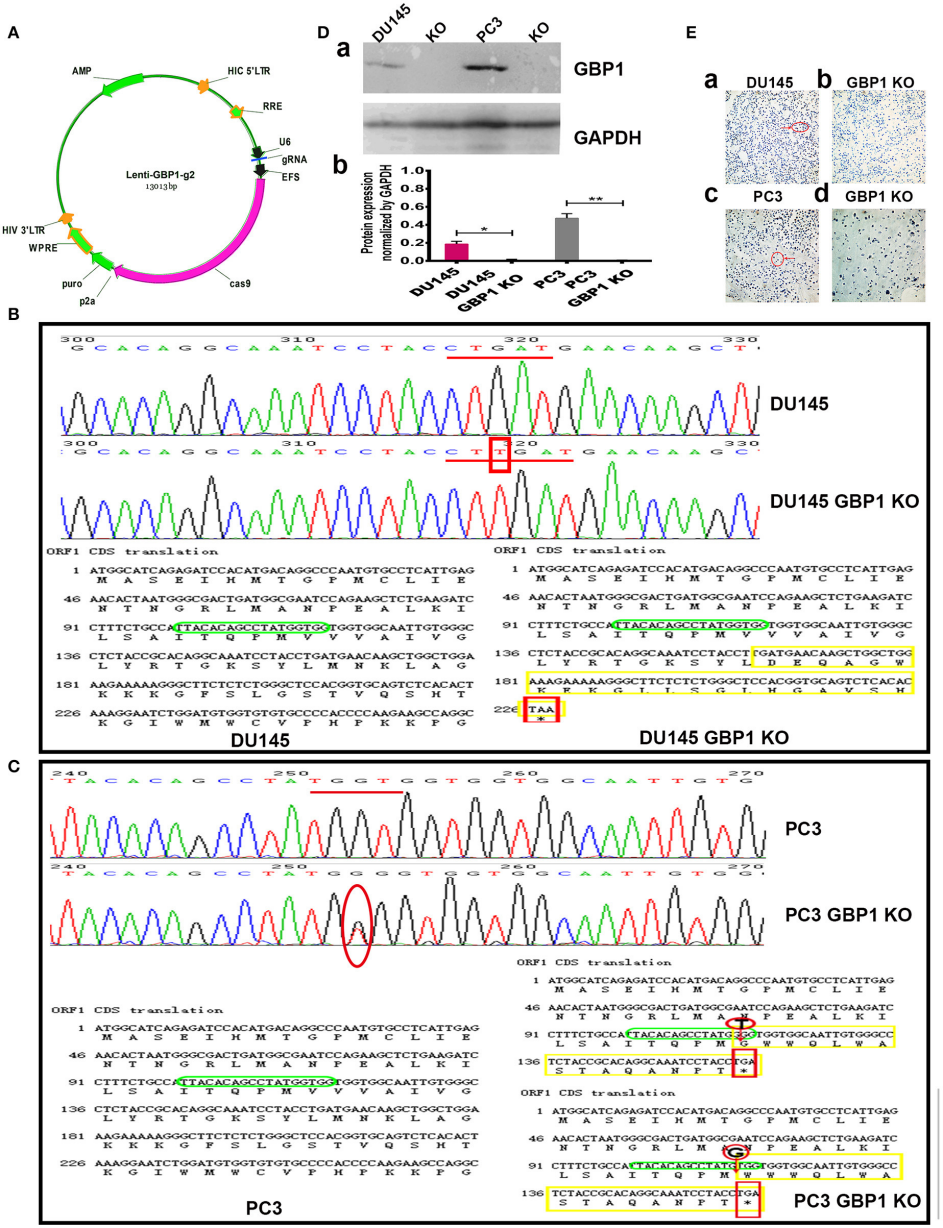
Generation and confirmation of DU145 GBP1 KO and PC3 GBP1 KO cells in the prostate cancer cell lines. **(A)** Shows the plasmid construction which was used in the process of CRISPR/Cas9 mediated GBP1 gene knockout. **(B)** The representative sequencings of GBP1 PCR products in the DU145 and DU145 GBP1 KO cells. The upper part shows the representative GBP1 PCR product sequencing charts of the DU145 and DU145 GBP1 KO cells, where a “T” insertion is marked in red. The lower part shows the corresponding cDNA sequences and the amino acid chains, in which, the “T” insertion resulted in mutated amino acid (yellow) followed by a termination code “TAA” (red). The sgRNA targeted sequence is marked in green. **(C)** Shows the representative sequencing charts of GBP1 PCR products in the upper part, where the mutation are marked in red circle, and the corresponding cDNA sequences and amino acid sequences are shown in the lower part where the mutations are marked in red circle, the stop code “TGA” is marked in red square and the mutated amino acids are marked in yellow. **(D)** Western blotting results of GBP1 protein expression in the control and GBP1 KO cells and corresponding densitometry histograms. **(E)** Shows the ICC results of control and GBP1 KO cells, and there is no GBP1 protein expression revealed by ICC in DU145 GBP1 KO or PC3 GBP1 KO cells (Magnification 200x).

To investigate the function of GBP1 gene in prostate cancer, two stable GBP1 KO cell lines were established from the DU145 and PC3 cells with CRISPR/Cas9 technology. The plasmid construction is shown in Figure 1A. Figure 1B shows the results of the PCR product sequencing results of the parental DU145 and DU145 GBP1 KO cell lines in the upper part, exhibiting a “T” insertion in both alleles in the second exon and the lower part shows parts of the corresponding CDS translations in the control and the GBP1 mutation cells flanking the mutation, where an early terminator (TAA) is marked in red, due to the “T” insertion-created frameshift in the DU145 GBP1 KO cells.

It was verified by PCR product sequencing that the PC3 GBP1 KO cell line harbored a “T” deletion in one allele and a “G” deletion in another allele in the second exon, compared to the control cell line (Figure 1C). The lower part shows parts of the corresponding CDS translations in the control and the GBP1 KO cells flanking the mutations. This mutations resulted in an early termination code TGA as shown marked in red square line. The KO status in both cell lines were repeatedly verified as shown in Figure 1D, where “a” shows a representative Western blot result, and “b” shows the corresponding histograms. GBP1 protein expression in the cell lines was further evaluated with ICC as shown in Figure 1E, where variable GBP1 expression is shown in the parental DU145 (1Ea) and PC3 (1Ec) cells, but the GBP1 protein expression in the DU145 GBP1 KO (1Eb) and PC3 GBP1 KO (1Ed) cell lines disappeared.

### GBP1 Gene KO Suppressed Prostate Cancer Cells Proliferation

**Figure 2:**
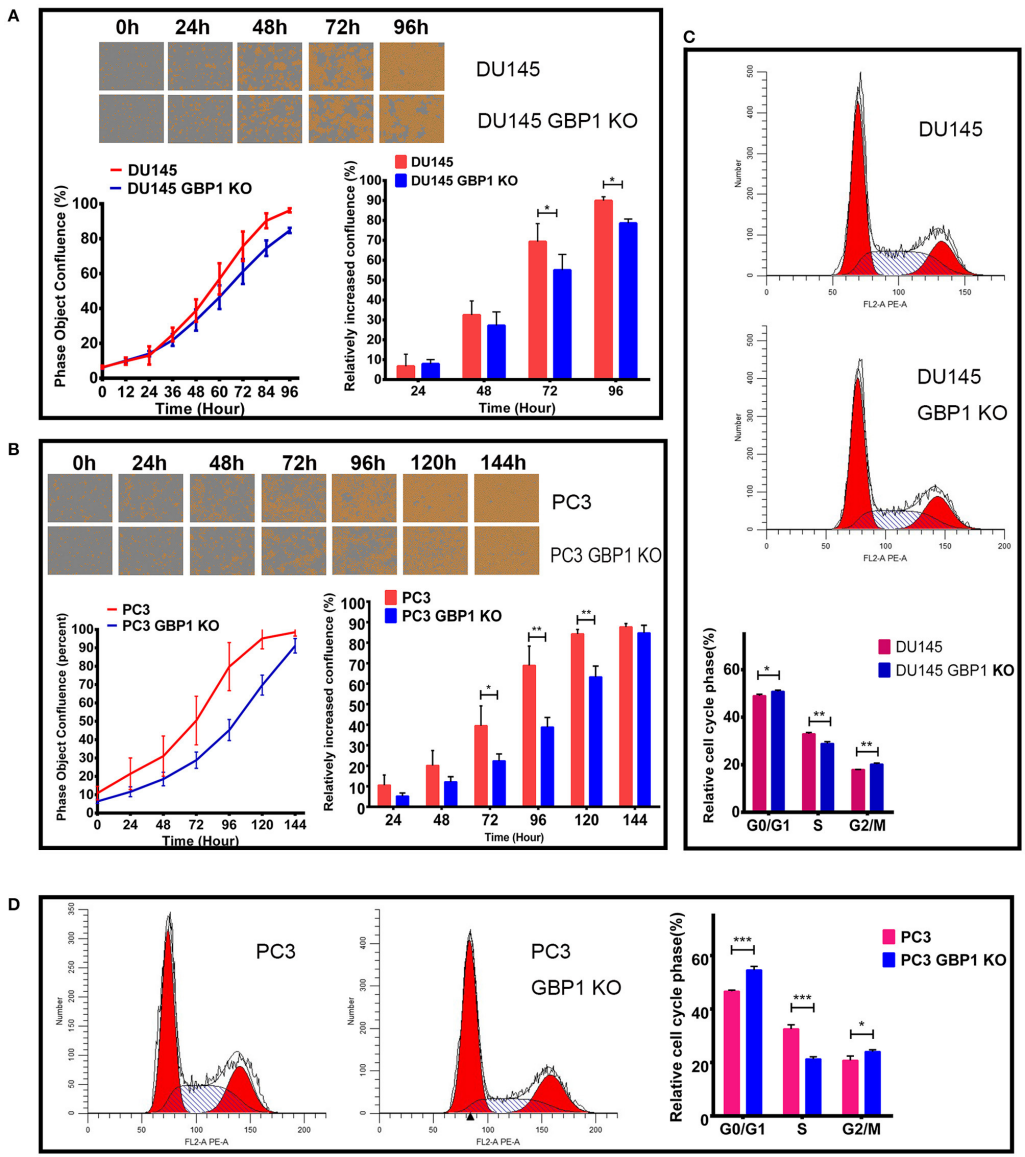
The characteristics of growth and cell cycles in the GBP1 KO cells. **(A)** and **(B)** show the real-time images and growth curves of the control and GBP1 KO cells both in DU145 and PC3 groups, which were generated by Incucyte ZOOM system. Both the DU145 GBP1 KO and PC3 GBP1 KO cells exhibit significant lower proliferation abilities compared with the control cells as shown in the histograms. Representative cell cycle analyses performed with flow cytometry for the DU145 and PC3 cells and corresponding histograms of the cell cycle distribution are shown in **(C,D)**, respectively. The data is presented as means from three independent experiments. Statistical significance: *p < 0.05, **p < 0.01, ***p < 0.001.

To determine whether the GBP1 gene KO had an effect on cancer cell growth, we firstly used the IncuCyte ZOOM System to record the real-time phase-contrast images and growth curves of different cell lines. It indicated that, there was no significant difference in cell morphology and cell proliferation ability between DU145 and DU145 GBP1 KO cells within the initial 48 h. However, significantly slower growth was noticed in the DU145 GBP1 KO cell line compared with the DU145 cells after 72 h (Figure 2A). For the PC3 and PC3 GBP1 KO cell lines, growth curves demonstrated that the growth of the GBP1 KO cells was suppressed compared with the control cells (Figure 2B), and the histogram exhibited a significant difference from 72 h in culture. For further analysis, the cell cycle distribution of these cancer cells was examined by flow cytometry. As shown in Figure 2C, the GBP1 gene KO induced higher percent of G0/G1 phase cells (*p* = 0.015) and lower percent of S phase cells (*p* = 0.002) in the DU145 cell line. In line with the DU145 cells, there was a significant increase in the percentage of G0/G1 phase (*P* < 0.001) and a decrease in the percentage of S phase (*P* < 0.001) in the PC3 GBP1 KO cells compared with the control group cells (Figure 2D).

### GBP1 KO Cells Were Revealed With Significantly Lower Migratory Potential

**Figure 3:**
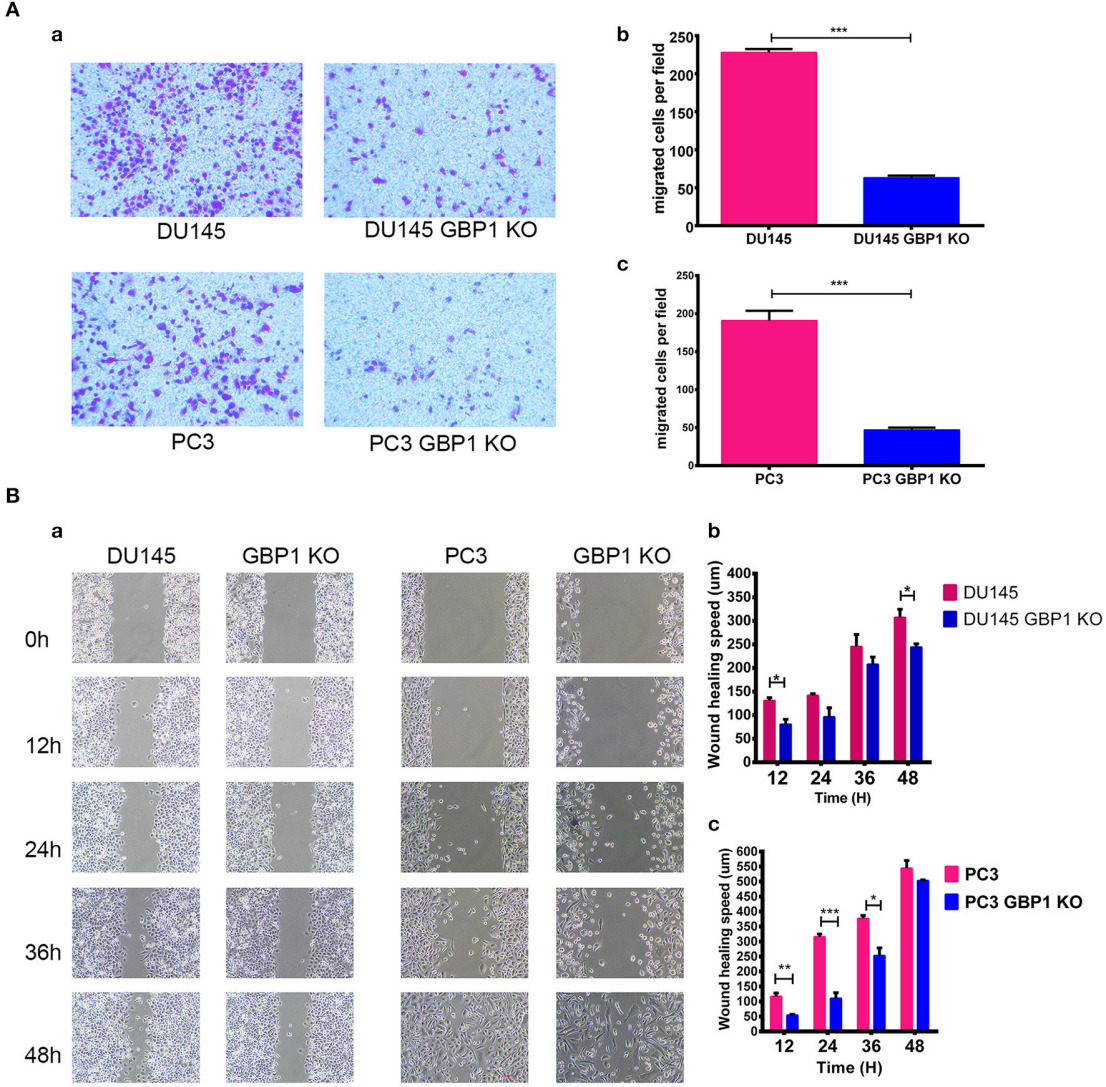
GBP1 KO cells displayed a significantly lower mobility. **(A)** Shows the results of cell motility assessed by transwell assays for DU145 cells and PC3 cells **(a)**, and the corresponding histograms are shown in **(b,c)**. The data is presented as means from three independent experiments and each experiment includes at least three parallel samples (mean ± S.D). **(B)** Shows the wound-healing images **(a)** and corresponding histograms for the DU145 cells **(b)** and PC3 cells **(c)**. The data are presented as means ± S.D (*n* = 3). Statistical significance: *p < 0.05, **p < 0.01, ***p < 0.001.

The transwell assay was used to evaluate the migration of the GBP1 KO and control cells. As shown in Figure 3A, significantly fewer GBP1 KO cells penetrated through the 8 μm diameter pore transwell membranes compared to the control cells (*P* < 0.001 for both cell lines). To further study the migratory ability alteration, wound healing assay was performed in the cells as well. The cells' healing speed was calculated (μm/12 h). Representative images are shown in Figure 3Ba. The DU145 GBP1 KO cells exhibited a significantly slower healing speed at 12 and 48 h in culture than the control cells (Figure 3Bb). Although no significant healing difference was observed in the PC3 GBP1 KO cells at 48 h, significantly slower healing speed was confirmed in these cells at 12, 24, and 36 h in culture (Figure 3Bc), compared to their parental control cells. All the above results demonstrated a role GBP1 gene cell migration and tumor progression in prostate cancer.

### GBP1 Gene KO Impaired Oxidative TCA Cycle

**Figure 4:**
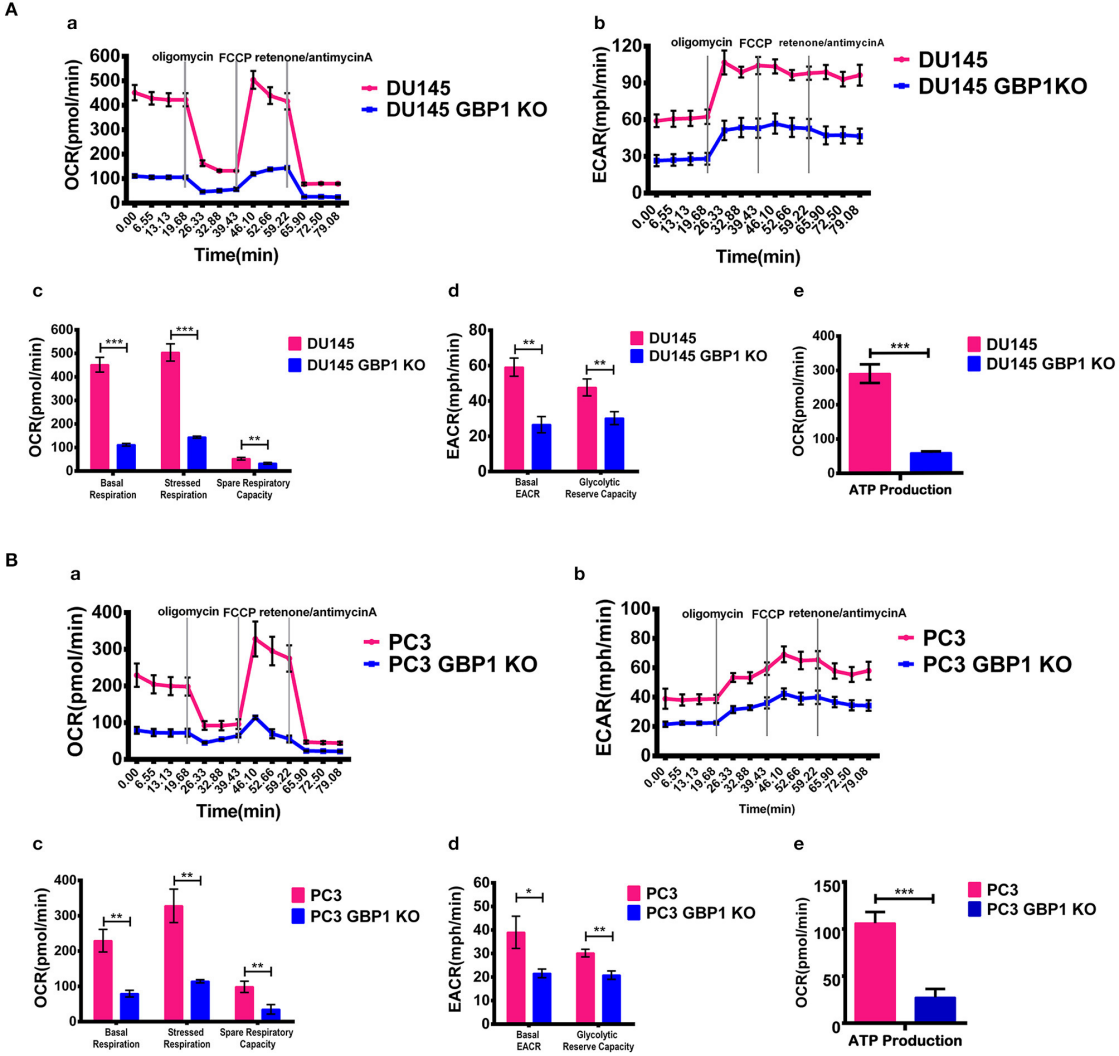
Results of mitochondria oxidative phosphorylation assay. **(A,B)** Show the results of mitochondria oxidative phosphorylation assays. Results of OCR and ECAR are shown in **(a,b)**, respectively. Histograms of OCR and ECAR are shown in **(c,d)**, respectively. Results of ATP production analysis are shown in **(e)**. The respiratory reserve capacity was calculated as the difference between basal and maximal, and the maximal OCR capacity was determined by the values stimulated by FCCP. ATP linked respiration was derived from the difference between OCR at baseline and respiration following oligomycin addition. Statistical significance: *p < 0.05, **p < 0.01, ***p < 0.001.

The previous studies have revealed that GBP1 knockdown impaired mitochondrial respiratory function, which was further supported by down-regulation of genes encoding electron transport chain components and genes involved in mitochondrial function (32). Therefore, we performed a cellular respiratory assay using the Seahorse XF Analyzer to assess mitochondrial function in the DU145 GBP1 KO and PC3 GBP1 KO cells. Oligomycin application was to block ATP synthesis by inhibiting ATP synthase, FCCP was to uncouple ATP synthesis from the flow of electrons in the electron transport chain (ETC) and rotenone + antimycin A was to block ETC complexes I and III, respectively. Our results showed that GBP1 KO cells had significantly decreased oxygen consumption rate (OCR) compared to control cells. GBP1 gene KO-induced impairment in mitochondrial respiration was pronounced in the DU145 GBP1 KO and PC3 GBP1 KO cells, as demonstrated by significant reductions in baseline respiratory rate, maximal respiration rate, coupling efficiency, and spare respiratory capacity (Figures 4Aa,Ac,Ba,Bc). Extracellular acidity rate is considered an indirect analysis of the glycolytic rate of cells, and glycolytic reserve capacity indicates the ability of a cell to perform glycolysis in response to an energetic demand. The DU145 GBP1 KO and PC3 GBP1 KO cells exhibited a lower basal ECAR and glycolytic reserve capacity compared with the control cells (Figures 4Ab,Ad,Bb,Bd). Additionally, the ATP production also significantly decreased in the GBP1 KO cells (Figures 4Ae,Be). Since high respiratory reserve capacity and ATP production are always linked to high mitochondrial fidelity, our results suggested that GBP1 gene KO impaired both mitochondrial oxidative phosphorylation and the compensatory glycolysis generally seen in cancer cells.

### GBP1 Gene KO Induced Higher Therapeutic Sensitivity

**Figure 5:**
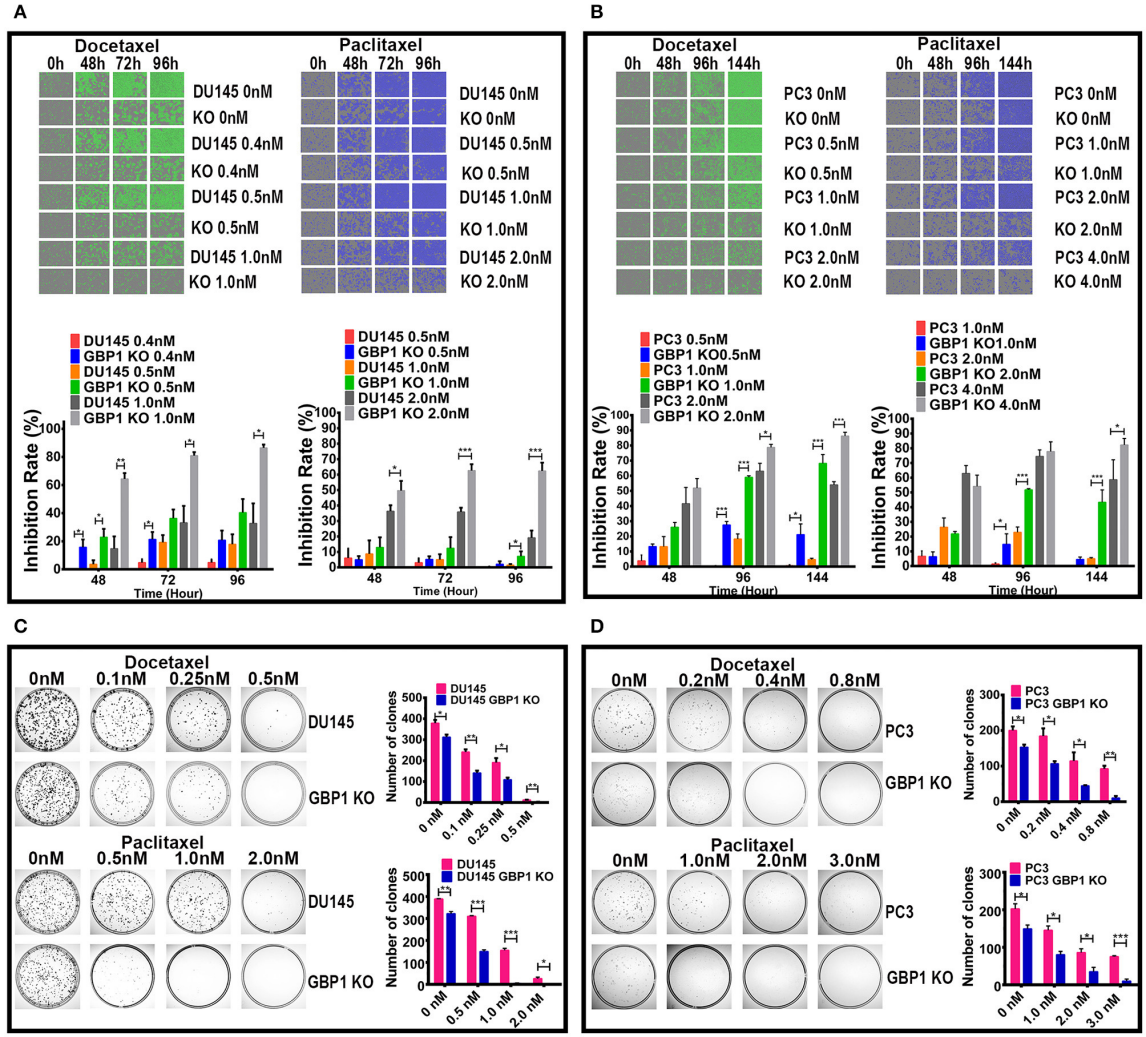
GBP1 KO cells were significantly more sensitive to paclitaxel and docetaxel. **(A,B)** Show real-time 2D color images and corresponding inhibition rate histograms of the DU145 and PC3 cells treated with docetaxel on the left and paclitaxel on the right, respectively. **(C,D)** Show the results of colony formation assay of the cells treated with docetaxel in the upper part, and the results of paclitaxel treatment in the lower part, respectively. The histograms of the colony formation assay results are shown on the right of **(C,D)**. The data is presented as means from three independent experiments (mean ± S.D). Statistical significance: *p < 0.05, **p < 0.01, ***p < 0.001.

Taxane drugs are commonly used chemotherapeutic agents to treat prostate cancer patients and some previous studies reported the positive correlation between GBP1 gene expression and docetaxel/paclitaxel resistance (26, 33, 34). To evaluate whether the GBP1 gene knockout affected the sensitivity of cancer cells to chemotherapy, we assessed cell proliferative ability with different concentrations of docetaxel and paclitaxel (Figures 5A,B) applied in cell culture. The drug optimum concentrations were chosen based on a series of different concentration of chemotherapeutic drugs in preliminary experiments, and the control cells were treated with equal amounts of solvent (DMSO). The growth rate and real-time phase contrast images were recorded by an IncuCyte ZOOM system. Regarding the docetaxel and paclitaxel treatment, a stronger concentration-dependent effect was observed in both DU145 and PC3 groups, including GBP1 KO and control cells. It exhibited a significantly suppressed growth capacity in the GBP1 KO cells compared with the control cells and proved the deletion of GBP1 gene resulted a significantly higher chemosensitivity of the cancer cells. For further study, the colony formation assay was used to analyze the cloning efficiency of the GBP1 KO and the control cells treated with different concentrations of docetaxel and paclitaxel. Representative photos of the colony formation assay for all the cell lines and corresponding histograms of the results are shown in Figures 5C,D. The number of clones was correspondingly decreased along with the increasing concentration of the chemotherapy drugs in both cell lines. In addition, the DU145 GBP1 KO and PC3 GBP1 KO cells exhibited a significantly lower colony efficiency than the control cells in all the drug concentration groups. Collectively, the above results implied that GBP1 gene KO plays an important role in chemotherapy sensitivity in prostate cancer cells.

### GBP1 Gene KO Decreased the Level of EGFR Protein Expression

**Figure 6:**
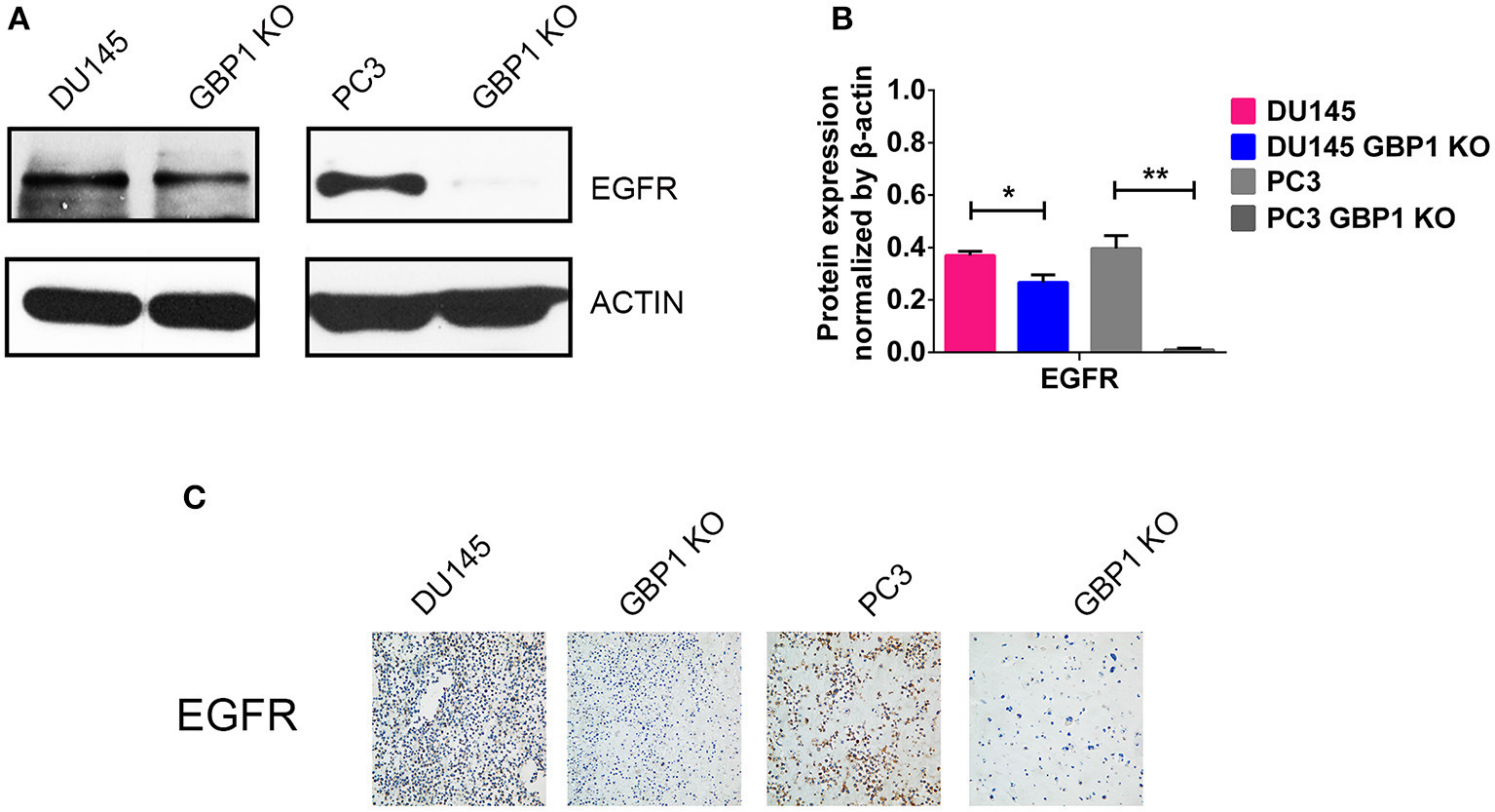
GBP1 KO cells exhibited significantly lower level TP53 and EGFR expression. **(A,B)** Show the Western blotting results of EGFR and corresponding histograms, respectively. **(C)** Shows ICC results of the EGFR in the DU145 and PC3 cells (magnification:200×). The data are presented as means ± S.D (*n* = 3). Statistical significance: *p < 0.05, **p < 0.01.

EGFR is a 170 kDa proto-oncogene and transmembrane receptor which is frequently overexpressed and has been associated with aggressive forms of PCa (35, 36). Ligand binding to EGFR induces dimerization, phosphorylation and internalization of the EGFR which then trigger a network of intracellular signaling pathways, resulting in DNA synthesis, cell proliferation, migration and adhesion (35). It also proved that EGFR promoted the survival of prostate tumor-initiating cells (TIC) and circulating tumor cells (CTC) that metastasize to bone (37). Some studies showed that EGFR was predominantly expressed in hormone-refractory and metastatic prostate cancer (38, 39). As we were working on EGFR and this gene was linked to chemotherapy-resistance and tumor malignancy, protein expression of this factor was further examined in these cells with both Western blot technology and ICC (Figure 6). It was verified that the protein expression of EGFR in both the DU145 GBP1 KO and PC3 GBP1 KO cells was significantly decreased, compared to the control cells.

### GBP1 Functional Blockade With NSC756093 in Prostate Cancer Cells Disclosed Similar Results

**Figure 7:**
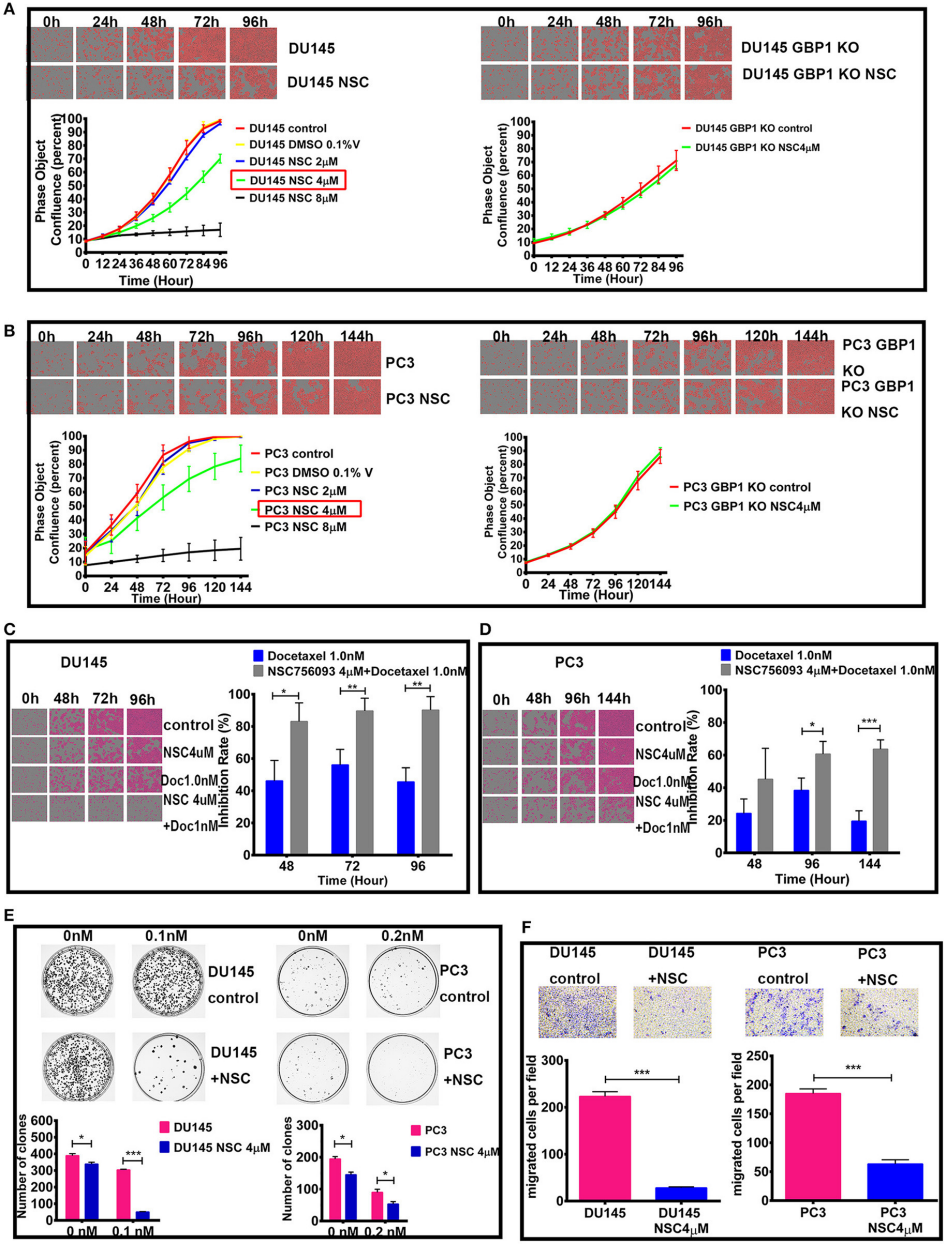
Effect of GBP1 blockade with NSC756093 on the DU145 and PC3 cells. The effects of different concentration of NSC756093 on control DU145 and PC3 cell growth are shown on the left part of **(A,B)**, respectively. The results of the DU145 GBP1 KO and PC3 GBP1 KO treatment effect with 4 μM NSC756093 are shown on the right part of **(A,B)**, respectively. **(C,D)** Show the real-time images and corresponding histograms of the cells treated with 1 nM docetaxel with or without 4 μM NSC756093 for the DU145 and PC3 cells, respectively. Representative modified colony formation assay results for docetaxel (0.1 nM for DU145 cell line and 0.2 nM for PC3 cell line) sensitivity with or without NSC756093 treatment are shown in **(E)**. Representative transwell assay images and corresponding histograms are shown in **(F)**. Statistical significance: *p < 0.05, **p < 0.01, ***p < 0.001. Data were expressed as mean ± S.D. Three replicated experiments were carried out with similar results.

NSC756093 is a potent *in vitro* inhibitor of the GBP1:PIM1 interaction and this property is maintained *in vivo* in ovarian cancer cells resistant to taxane (31). In the current study, we treated the DU145 and PC3 prostate cancer cells with 4 μM NSC756093 to further study the function of GBP1 in regulation tumor cell proliferation, migration and drug resistance after initial dose optimization. NSC756093 was added into the culture medium of DU145 and PC3 cells, and the cells all grow slowly under the inhibitor application as shown in Figures 7A,B. However, when NSC756093 was applied in DU145 GBP1 KO and PC3 GBP1 KO cell lines, there was no apparent decrease in growth curve. To further explore whether the NSC756093 blockade could mimic GBP1 gene KO, 4 μM NSC756093 with or without 1 nM docetaxel were examined in the control cells for chemosensitivity analyses, which revealed higher docetaxel sensitivity when the NSC756093 was applied together in both DU145 (Figure 7C) and PC3 cells (Figure 7D). The NSC756093 influence on docetaxel sensitivity was also additionally tested in modified colony formation assay, with or without the combinational use, which also verified that both DU145 and PC3 cells were significantly more sensitive to the docetaxel (Figure 7E). The migration ability of the DU145 and PC3 cells were also significantly reduced after the GBP1 blockade NSC756093 application (Figure 7F).

### GBP1 Gene KO Inhibited Xenograft Growth of Prostate Cancer Cells

**Figure 8:**
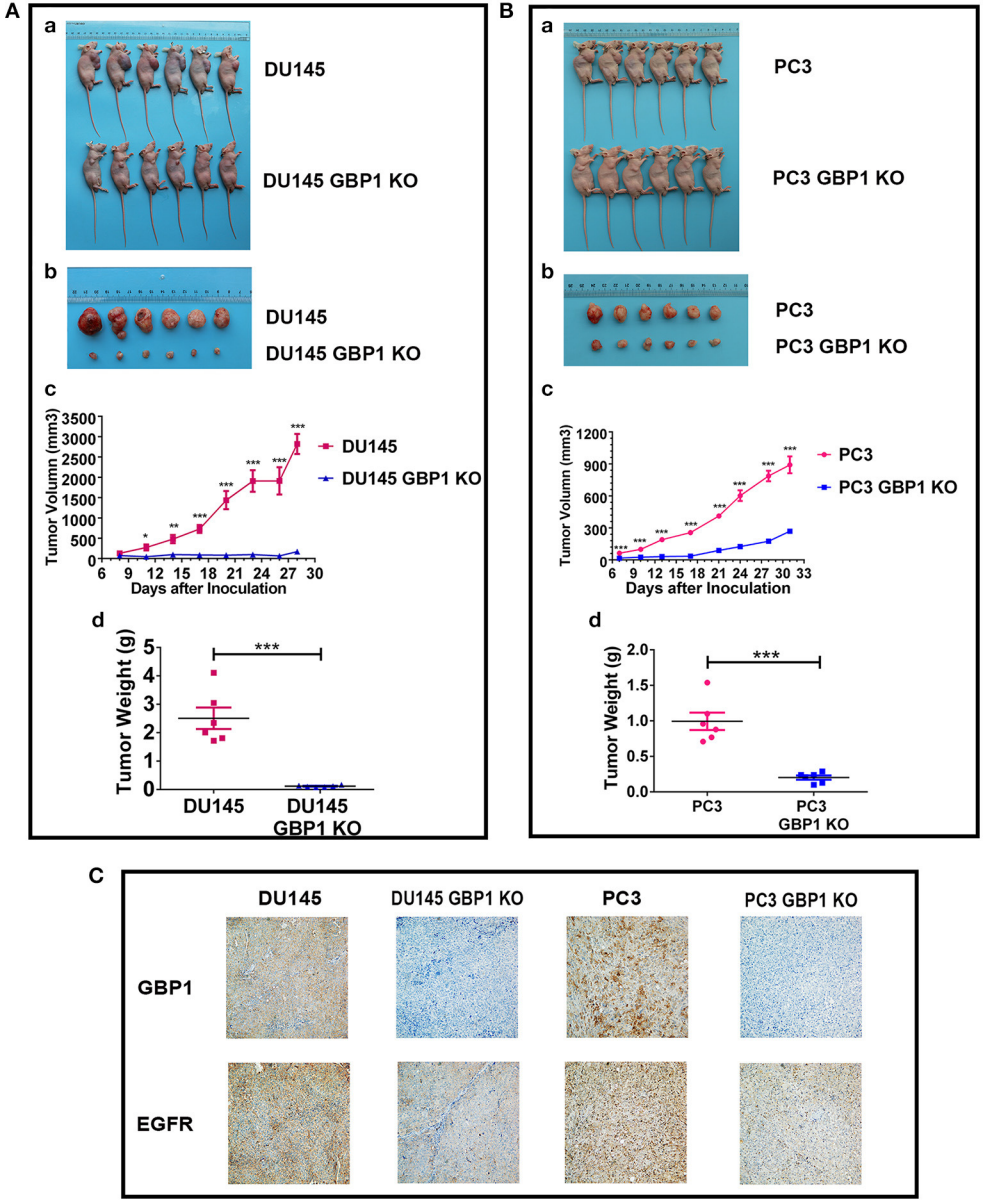
GBP1 gene KO inhibited tumor growth *in vivo*. **(A,B)** Show the results of the effects of GBP1 gene KO on xenograft growth. **(a)** and **(b)** Show the images of BALB/c mice and xenograft tumors, respectively. Tumor volume and tumor weight o of the GBP1 KO and control cells are shown in **(c)** and **(d)**, respectively. **(C)** Shows the results of IHC of GBP1, EGFR on the xenograft tumors (Magnification 200x).

Before the experiment, cell number and the whole experiment procedure were optimized with the control PC3 and DU145 cells. It was confirmed in the current study that GBP1 KO cells in this xenograft model grew very slow compared with the control parental cells in both groups (Figure 8). Comparatively, GBP1 KO xenograft tumor volume over time in both groups was significantly smaller than that in the corresponding control group (*p* < 0.001 for the DU145, and *p* < 0.001 for the PC3 cells). Similarly, significantly reduced tumor weight in the GBP1 gene KO xenografts in both groups was verified in comparison to their control tumors (Figures 8A,B). Immunohistologically (Figure 8C), GBP1 protein expression in the xenograft tumors disappeared while the control xenograft tumors in both DU145 and PC3 groups were GBP1 positive. The protein expression level of EGFR was decreased in the GBP1 KO xenograft tumors compared with the tumors of control cells.

### Increased GBP1 Protein Expression Was Correlated With Poor Clinicopathological Characteristics

**Figure 9:**
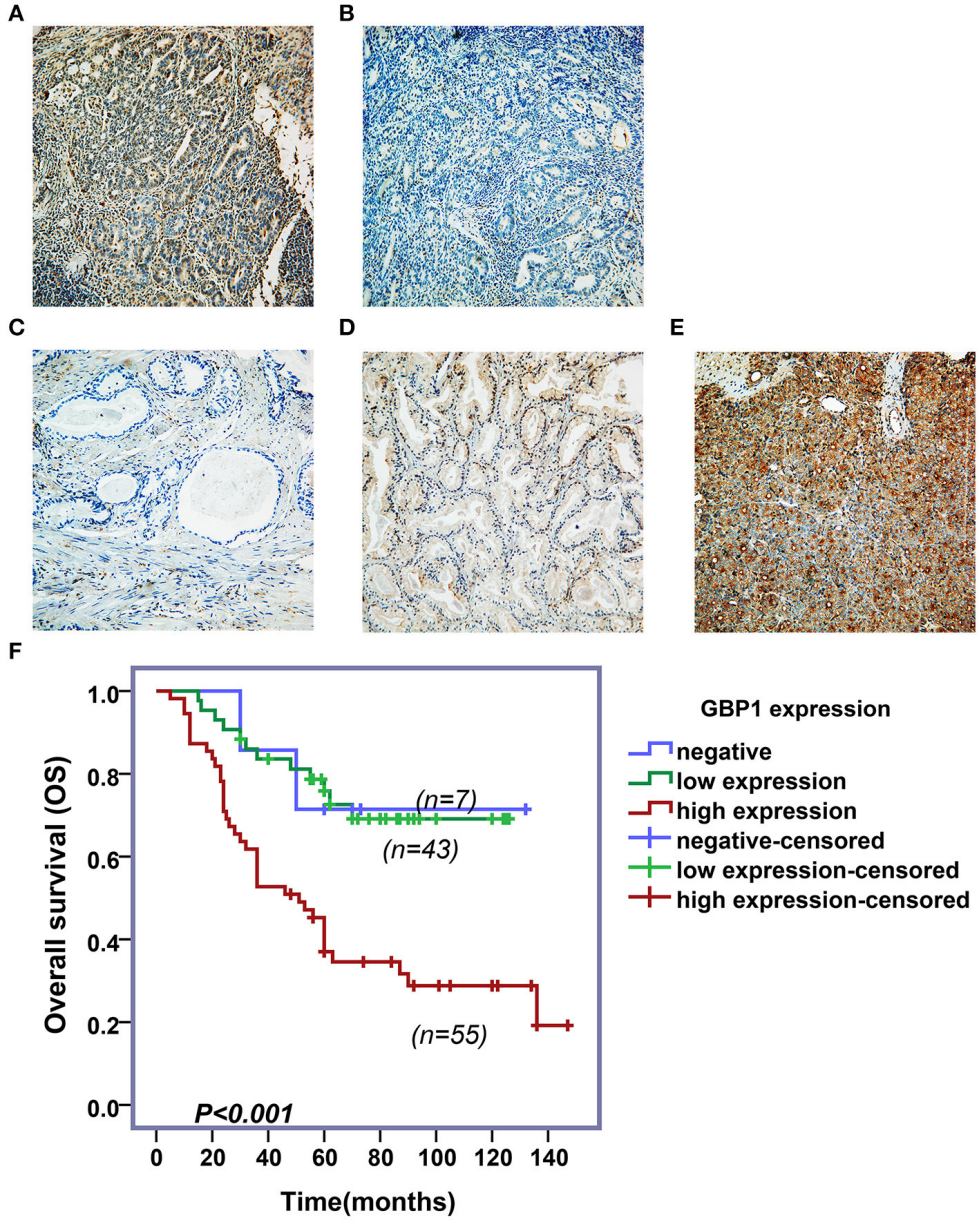
Immunohistochemical assay and survival analyses. Variable levels of typical diffuse GBP1 cytoplasmic and membranous staining is shown in the PCas. Positive and negative controls are shown in **(A,B)**, respectively. Tumors with negative, moderate and strong GBP1 protein expression are shown in **(C–E)**, respectively. All images are taken at 200x. Results of Kaplan–Meier survival plotting is shown in **(F)**, where high levels of GBP1 protein expression exhibit significantly positive association with a shorter overall survival in PCa (*P* < 0.001).

To investigate the association of GBP1 protein expression and survival in prostate cancer patients, expression of GBP1 protein in a series of 105 PCa clinical samples with survival data were detected by IHC. Typical immunostained slides with different levels of GBP1 expression in cytoplasm and cytomembrane are shown in Figure 9. It was discovered that 7 (6.67%) samples were negative, 43 (40.95%) were mildly positive and other 55 (52.38%) were highly positive for the expression of GBP1 protein. The association between GBP1 expression and the clinicopathological features were analyzed, and the summarized in Table 2. It was discovered that GBP1 protein expression was significantly associated with higher Gleason score (p = 0.009), and distant metastasis (*p* = 0.012). No significant association was found between the GBP1 protein expression and other clinical parameters such as age, PSA and TNM stage. The overall survival (OS) curve was calculated using the Kaplan-Meier method and compared by the log-rank test. The follow-up period ranged from 2 to 147 months with a median overall survival time 58 months. High GBP1 protein expression in these PCa tissues was significantly associated with poor overall survival (*P* < 0.001; Figure 9F).

**Table 2:** Correlations between tumor GBP1 expression and clinical variables.

**Variable**	**Total**	**GBP1-Expression (*****n%*****)**			
		**(–)**	**(+)**	**(++)**	***P-*Value^a^**
Age (years)	105				
<66	32	1 (3.1)	15 (46.9)	16 (50.0)	0.412
66–75	39	5 (12.8)	15 (38.5)	19 (48.7)	
>75	34	1 (2.9)	13 (38.2)	20 (58.8)	
PSA (ng/ml)					
<10	14	1 (7.1)	5 (35.7)	8 (57.1)	0.990
10–100	45	3 (6.7)	19 (42.2)	23 (51.1)	
>100	46	3 (6.5)	19 (41.3)	24 (52.2)	
Gleason score					
<7	33	1 (3.0)	18 (54.5)	14 (42.4)	**0.009**
7	43	6 (14.0)	18 (41.9)	19 (44.2)	
>7	29	0 (0.0)	7 (24.1)	22 (75.9)	
TNM stage					
T1/T2	78	3 (3.8)	36 (46.2)	39 (50.0)	0.070
T3/T4	27	4 (14.8)	7 (25.9)	16 (59.3)	
Lymph node metastasis					
Negative	89	6 (6.7)	38 (42.7)	45 (50.6)	0.760
Positive	16	1 (6.3)	5 (31.3)	10 (62.5)	
Distant metastasis					
Negative	76	2 (2.6)	36 (47.4)	38 (50.0)	**0.012**
Positive	29	5 (17.2)	7 (24.1)	17 (58.6)	

a *Pearson Chi-Square test. Bold indicates p < 0.05*.

In addition, univariate and multivariate analyses were performed by using Cox proportional hazards regression method to determine independent prognostic factors for overall survival in PCa patients. As shown in Table 3, our results indicated that GBP1 expression, Gleason score, and TNM stage had a correlation between the overall survival in the univariate analysis. Therefore, these above three parameters met the criterion (*P* < 0.05) for the multivariate analysis, and the multivariate analysis identified all these three as independent risk factors for shorter OS in the PCa patients as well.

**Table 3:** Univariate and multivariate analysis for overall survival using COX relative risk.

**Variable**	**Univariate analysis**			**Multivariate analysis**		
	**HR**	**95% CI**	***P*-value^a^**	**HR**	**95% CI**	***P*-value^a^**
GBP1 expression	2.630	1.518–4.556	0.001^**^	2.575	1.455–4.558	0.001^**^
Gleason score	2.257	1.546–3.297	<0.001^***^	1.789	1.194–2.680	<0.001^***^
TNM stage	2.015	1.123–3.618	0.019^*^	2.022	1.115–3.669	0.018^*^
Clinical stage	1.381	0.799–2.386	0.248			
Age	1.319	0.933–1.866	0.117			
PSA	0.758	0.516–1.115	0.160			
Lymph node metastasis	0.970	0.436–2.157	0.941			
Distant metastasis	1.012	0.553–1.850	0.970			

a *Cox regression. Bold indicates p < 0.05*.

*HR, relative risk; 95% CI, 95% confidence interval*.

Statistical significance: ^*^p < 0.05, ^**^p < 0.01, ^***^p < 0.001.

## Discussion

In the earlier studies, GBP1 was mainly regarded as a interferon/cytokine inducer or virus infection responder in hostcells (1, 2, 8, 10, 12–16). GBP1 came to our research focus only after we systemically treated the prostate cancer cell line DU145 with ethidium bromide and flavopiridol for a long period and studied the molecular mechanism behind how those cells survived such treatments. The common features for those cancer cells survived such treatments are as the followings: the DU145 cancer cells under the treatments experience a sharp decline of growth/cell number during the first 1–2 weeks, then singular cells can be identified as living cells with extremely slow growth rate for a few weeks, and after this period, these tumor cells gradually recover their growth ability and can be maintained with the medium with the same concentration of ethidium bromide or flavopiridol for many months without noticeable change in morphology, growth ability and molecular features, Such tumors cells are revealed metabolic reprogramming with typical Warburg effect and chemo- and radiotherapy resistance (29, 30). Transcriptome analyses of these cells disclose significantly higher levels of GBP1 gene expression compared to their parental DU145 cells, which could be immunocytochemically verified with the cytoblocks (Supplemental Figure 1). This attempted us to explore the possible role of GBP1 in prostate cancer in this study.

To explore the possible role of GBP1 in prostate cancer, stable DU145 GBP1 KO and PC3 GBP1 KO cell lines were established using the CRISPR/Cas9 technology, in which one nucleotide mutation and early terminator were created in exon2 in both cell lines. The GBP1 KO prostate cancer cells grew significantly slower both *in vitro* and in xenograft models. In a series of *in vitro* studies, the GBP1 KO prostate cancer cells were significantly sensitive to both docetaxel and paclitaxel. We further studied the general cell biology features of these cells compared to their parental cells. It was verified that both the DU145 GBP1 KO and PC3 GBP1 KO cells showed significantly lower colony formation and wound healing abilities. Energy metabolism study with the Seahorse System revealed that both OCR and ECAR in the DU145 GBP1 KO and PC3 GBP1 KO cells were significantly inactivated, indicating strongly not only suppressed mitochondrial oxidation and oxidative phosphorylation function, but also significantly suppressed glycolysis in these cells, a typical “anergy” status. All these experiments revealed significantly less aggressive features in the DU145 GBP1 KO and PC3 GBP1 KO cells. In line with the *GBP1* gene knockout, the expression of the oncoprotein EGFR in the DU145 GBP1 KO and PC3 GBP1 KO cells was also reduced. Importantly, the DU145 GBP1 KO and PC3 GBP1 KO cells grew significantly slow in the xenograft model.

To evaluate efficacy difference of the GBP1:PIM1 interaction inhibitor NSC756093 (31) in these cells, the optimized concentration of NSC756093was applied in these GBP1 KO cells, with the parental cells as controls. As shown in Figure 8, there was no proliferation inhibition effect in the GBP1 KO cells, while the parental DU145 and PC3 cells showed significantly reduced proliferation rate after the NSC756093 application. These results largely support the role of NSC756093 inhibitor in prostate cancer cells, where GBP1 plays an important role as disclosed earlier (25, 40).

However, it was indicated by big data analysis that GBP1 could be associated with better survival in prostate cancer (41). By carefully analyzing the study it is know that the data was collected from clinical sample transcriptome measurements. As it is discovered in our study that the GBP1 protein expresses variably in non-tumor cells like fibroblasts and lymphocytes (Supplemental Figure 2), and the RNA level GBP1 expression in non-tumor cells exists obviously. Heterogenous tumor cell expression and tumor cell percentage in a given case are the other two issues, for which general gene expression profile data should be explained with care as well, and further immunohistochemical protein expression in large scale clinical samples is required to verify such findings.

Collectively, our current study supports the notion that GBP1 is a oncoprotein in prostate cancer, and high levels of GBP1 protein expression is significantly associated with aggressive features in cell line models *in vitro* and associated with malignant features and poor overall survival in clinical samples. This finding is largely in line with a series of cancer studies where oncogenic function of GBP1 is indicated in prostate cancer (25), triple negative breast cancer (23), esophageal squamous cell carcinoma (19), glioblastoma (17, 42, 43), ovarian cancer (20, 24, 26, 28), lung cancer (21) and oral cancer (18), and higher levels of GBP1 expression have been associated with enhanced tumor cell infiltration, metastasis, therapeutic resistance and shorter survival in these studies (18, 20, 21, 23, 25, 27, 42).

However, there are also opposite findings in GBP1 studies. An earlier study indicated a role of proliferation inhibition of GBP1 in intestinal epithelial cells through suppression of beta-catenin/T cell factor signaling (44). GBP1 suppressor role in colon cancer was also reported (22, 45–47) and reviewed (9). In the study reported by Britzen-Laurent et al., GBP1 positive expression was discovered mainly in stroma cells of colon cancer, and a large part of the colon cancer samples were negative for its expression, and their *in vitro* and *in vivo* studies demonstrated tumor suppressor role of GBP1 in colon cancer cells (22). In addition, the antiangiogenic effects of inflammatory cytokines in endothelial cells have been shown to be mediated by GBP1 (48–51). In hepatocellular carcinoma, low expression of the GBP1 gene in the non-tumorous tissue of the remnant liver was reported to be associated with early recurrence after surgical resection (52). However, it was also reported that even in the HepG2 cells, a clinically relevant radioresistant cell line, with naturally low GBP1 expression, knockdown of *GBP1* still could reduce the radioresistance shown by the parental cells (27). Contradictory findings regarding the function of GBP1 in breast cancer also exist. In an earlier murine mammary carcinoma transplantation model study, it was disclosed that interferon gamma-induced human GBP1 could inhibit the tumor growth (53), although the recent studies indicated its role of oncogene in breast cancer (23, 54). Through an *in vitro* blood-brain barrier model study, Mustafa et al. discovered that co-culture of breast cancer cells and activated T cells *in vitro* upregulated the GBP1 expression, and the GBP1 upregulation in the breast cancer cells facilitated their brain metastasis (54), a finding in line with our current study.

In general, the above contradictory findings in cancer studies may attribute to many factors, including methodologies applied, sample volume limitation and cancer type specificity of GBP1 role. It is now known that GBP1 expression is not only virus/cytokine inducible, but chemotherapy-created stress is also inducible for its expression, indicating a possible complex GBP1 expression in a given tumor where areas of tumor cells may exist in significantly different microenvironment niches. Therefore, carefully dissecting the patterns of GBP1 expression in large series of clinical tumor samples will help to identify its role in cancer and provide us clue for further targeting strategies.

In summary, our current study has demonstrated a pro-survival or oncogenic role of GBP1 in two independent prostate cancer lines by application of CRISR/Cas9 gene knock out technology. The GBP1 gene KO DU145 and PC3 prostate cancer cells showed significantly less aggressive *in vitro*, i.e., less proliferation, infiltration, wound healing and colony formation capabilities with a prominent “anergy” status in both mitochondrial oxidative phosphorylation and glycolysis, and with a significantly higher sensitivity to chemotherapeutic reagents. The xenograft experiments verified a significantly slower tumor growth of the GBP1 KO cells in nude mouse model. Furthermore, clinical prostate cancer sample GBP1 protein expression revealed its aggressive clinical feature correlation and shorter overall survival association.

## Data Availability Statement

All datasets generated for this study are included in the article/Supplementary Material. The raw data supporting the conclusions of this manuscript will be made available by the authors, without undue reservation, to any qualified researcher.

## Ethics Statement

All animal protocols were performed in accordance with the recommendations of the Institutional Animal Care and Use Committee of The First Affiliated Hospital of Zhengzhou University. The study was approved by the Ethics Committee of The First Affiliated Hospital of Zhengzhou University. All the patients involved provided written informed consent.

## Author Contributions

ZS, HL, and HF designed and coordinated the research. JZ and ZS mainly completed and drafted the manuscript. XL, LL, and JC assisted with the research and analyzed the data. MG provided substantial contributions to the conception of the manuscript and helped draft the manuscript.

### Conflict of Interest

The authors declare that the research was conducted in the absence of any commercial or financial relationships that could be construed as a potential conflict of interest.

## Supplementary Material

Supplemental Figure 1 Immunohistochemistry verification of GBP1 protein expression in prostate cancer cell lines. The control DU145 cells are shown weakly positive for the GBP1 antibody, while both ethidium bromide and flavopiridol treated DU145 cells are shown strong positive for the GBP1 antibody (x200).

Supplemental Figure 2 Immunohistochemical result of positive GBP1 protein expression in stromal cells of a prostate cancer. Areas of stromal cells (arrows) in a prostate cancer are shown with strong GBP1 protein expression, while the prostate cancer cells are largely negative for GBP1 protein expression (x200).

## Abbreviations

GBP1: guanylate binding protein 1
KO: knockout
IHC: Immunohistochemical
ICC: Immunocytochemistry.
