# Supplementary figures and images for: Oncogenic Role of Guanylate Binding Protein 1 in Human Prostate Cancer
Source: Front Oncol. 2020 Jan 10;9:1494. doi: 10.3389/fonc.2019.01494 (PMC6967410; doi:10.3389/fonc.2019.01494)

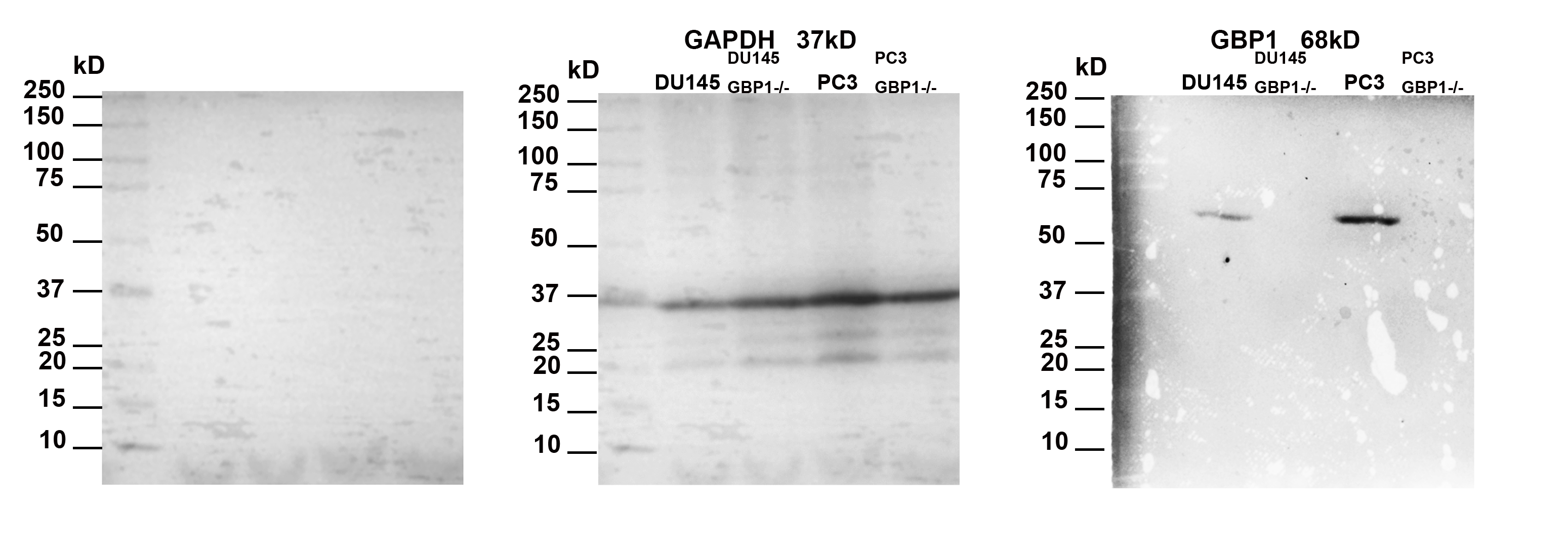

Supplement: Supplementary file 1 [file Data_Sheet_1.ZIP › raw data/figure 1/GBP1 WB╕▒▒╛.tif]

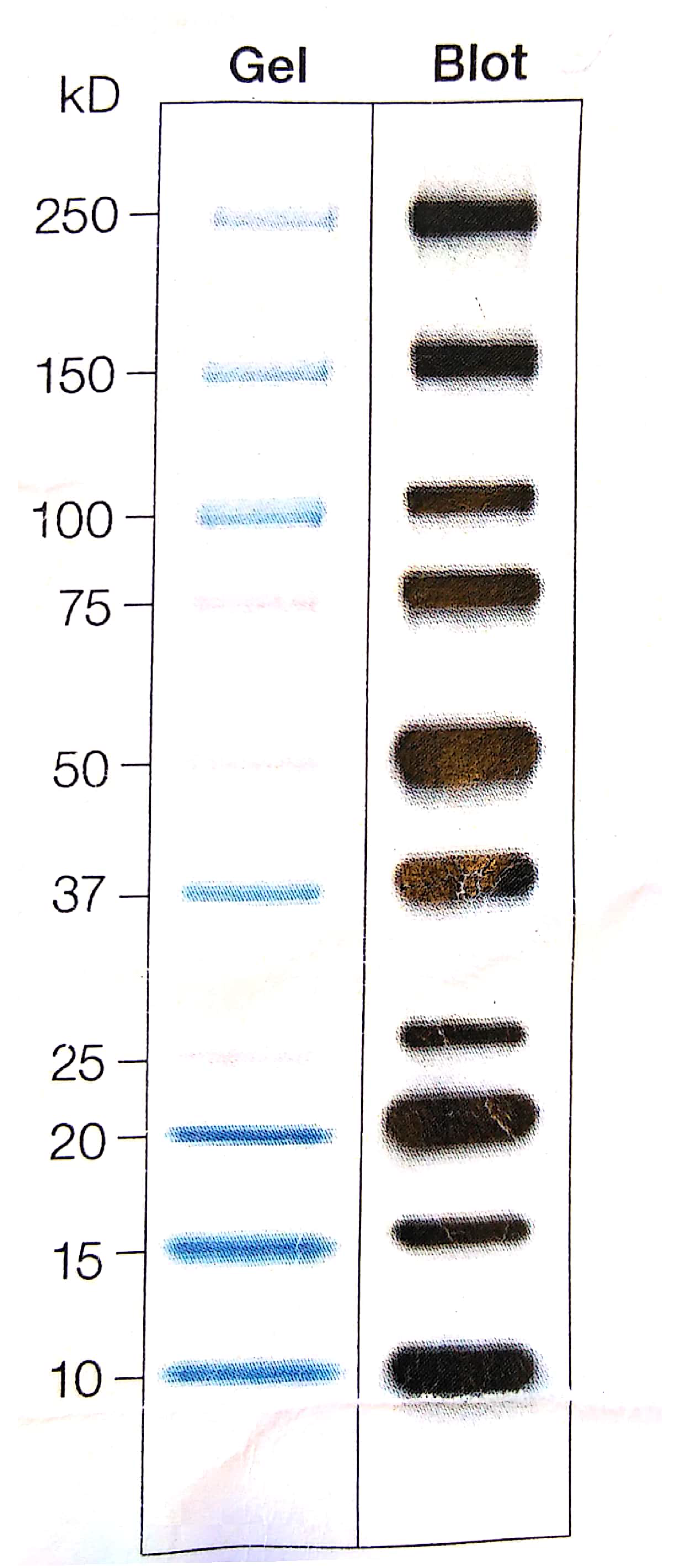

Supplement: Supplementary file 1 [file Data_Sheet_1.ZIP › raw data/figure 1/marker.tif]

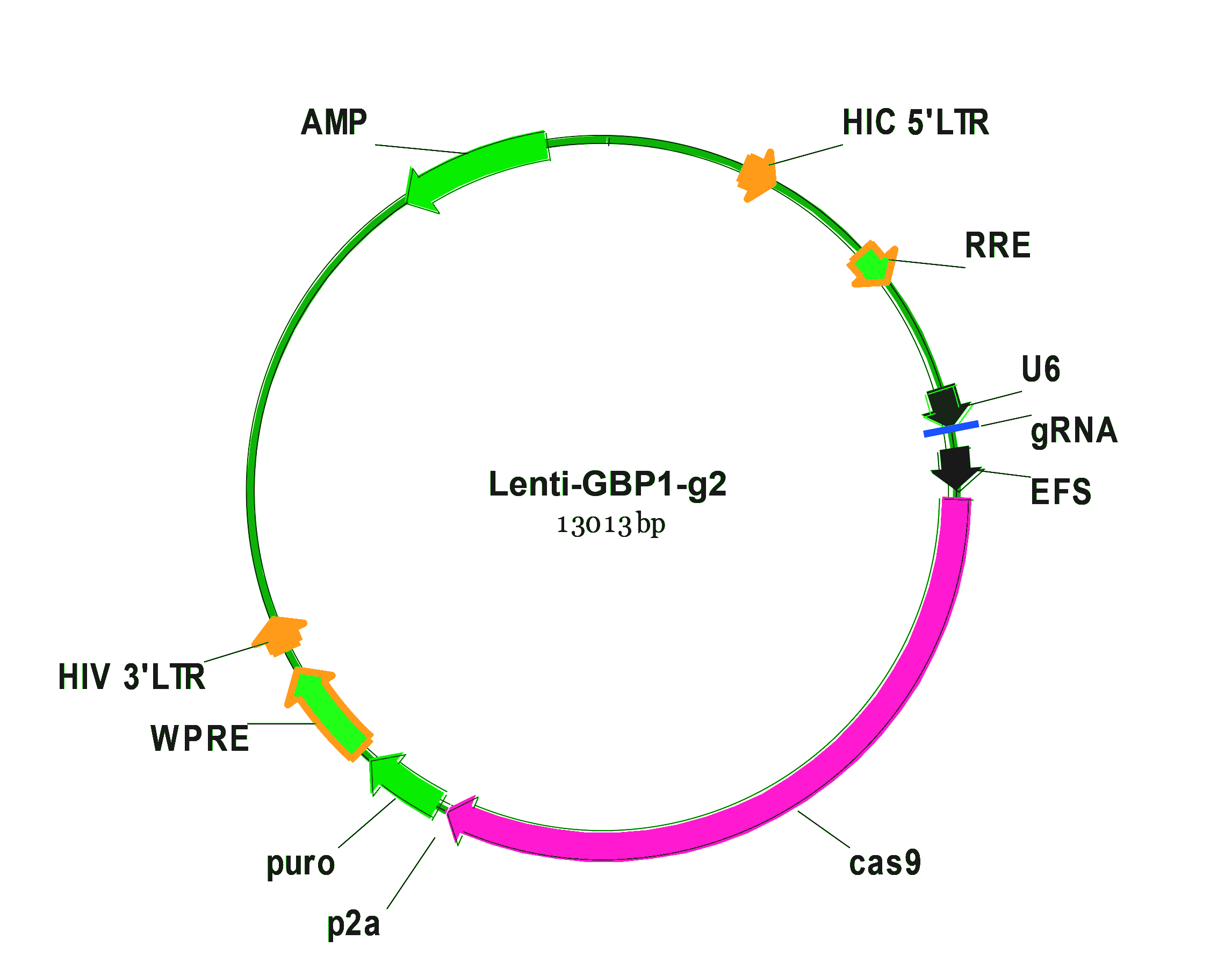

Supplement: Supplementary file 1 [file Data_Sheet_1.ZIP › raw data/figure 1/╓╩┴ú╗╖.tif]

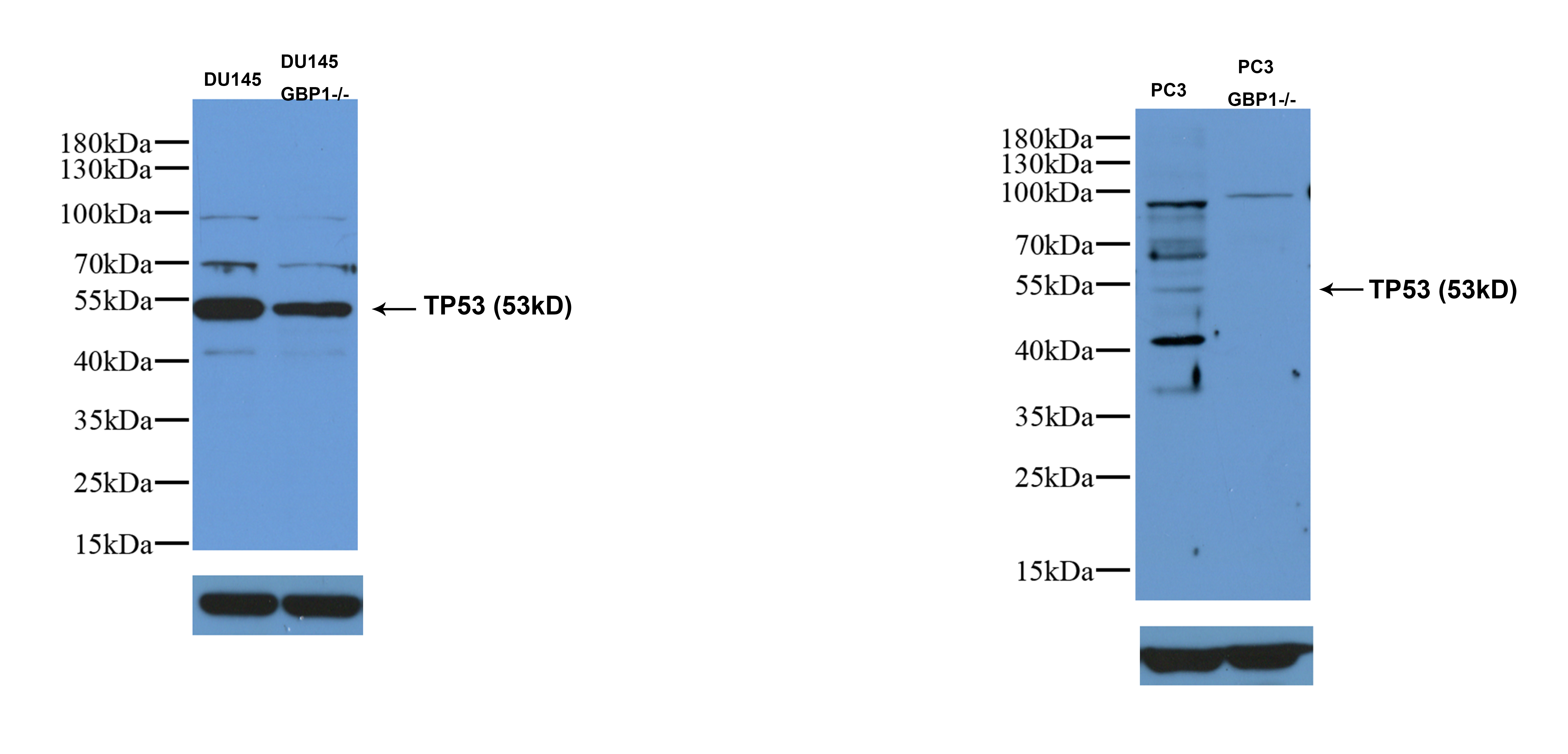

Supplement: Supplementary file 1 [file Data_Sheet_1.ZIP › raw data/figure 6/TP53╚1⁄2─ñ.tif]

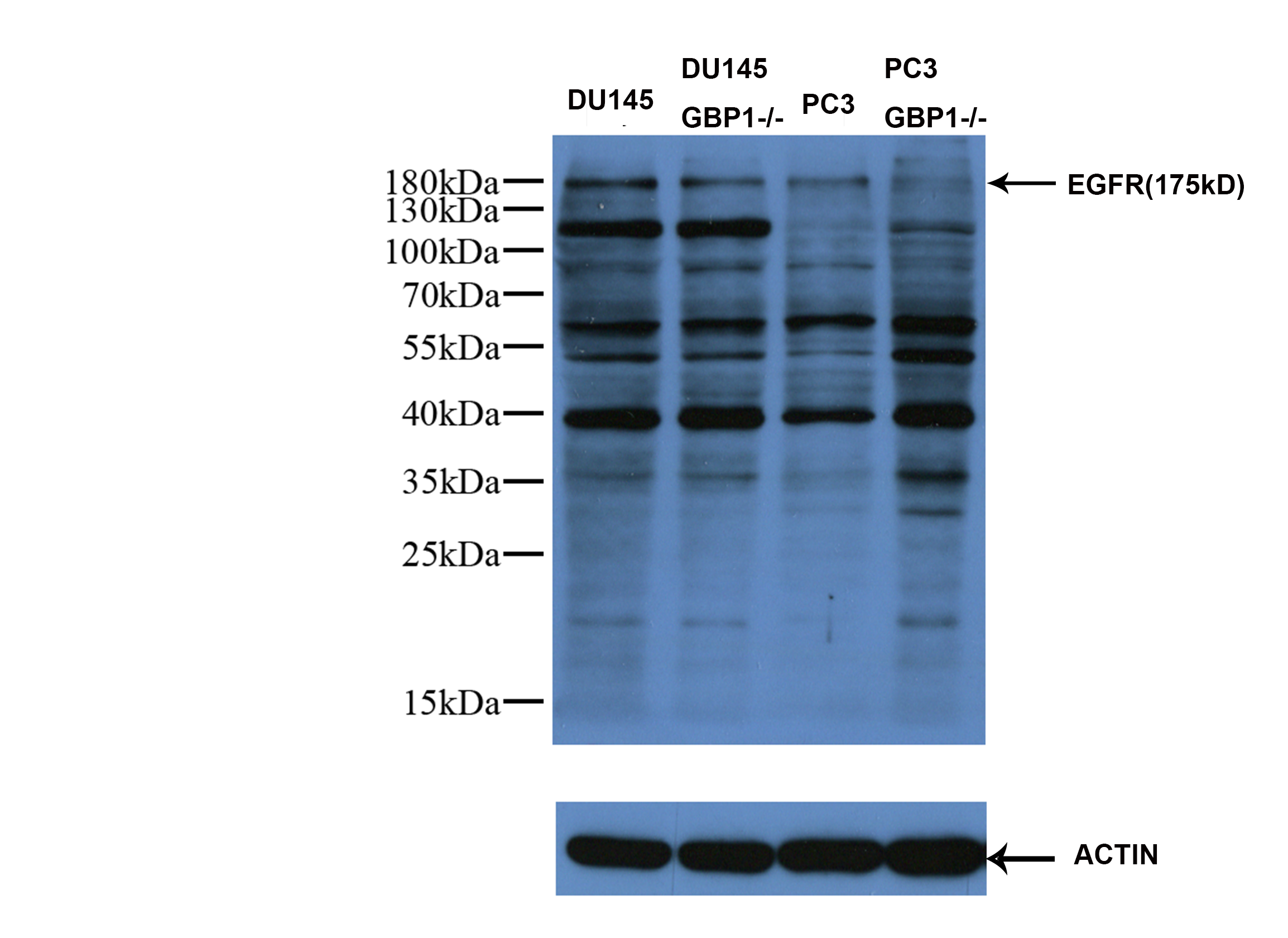

Supplement: Supplementary file 1 [file Data_Sheet_1.ZIP › raw data/figure 6/egfr-wb.tif]

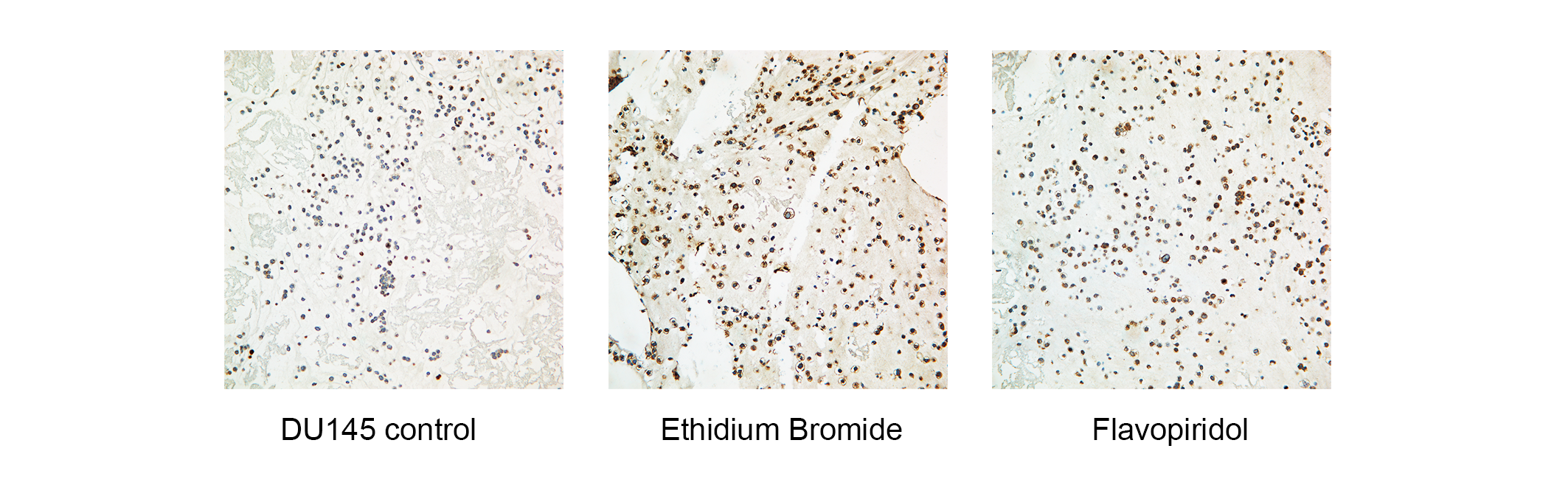

Supplement: Supplemental Figure 1 — Immunohistochemistry verification of GBP1 protein expression in prostate cancer cell lines. The control DU145 cells are shown weakly positive for the GBP1 antibody, while both ethidium bromide and flavopiridol treated DU145 cells are shown strong positive for the GBP1 antibody (x200). [file Image_1.TIF]

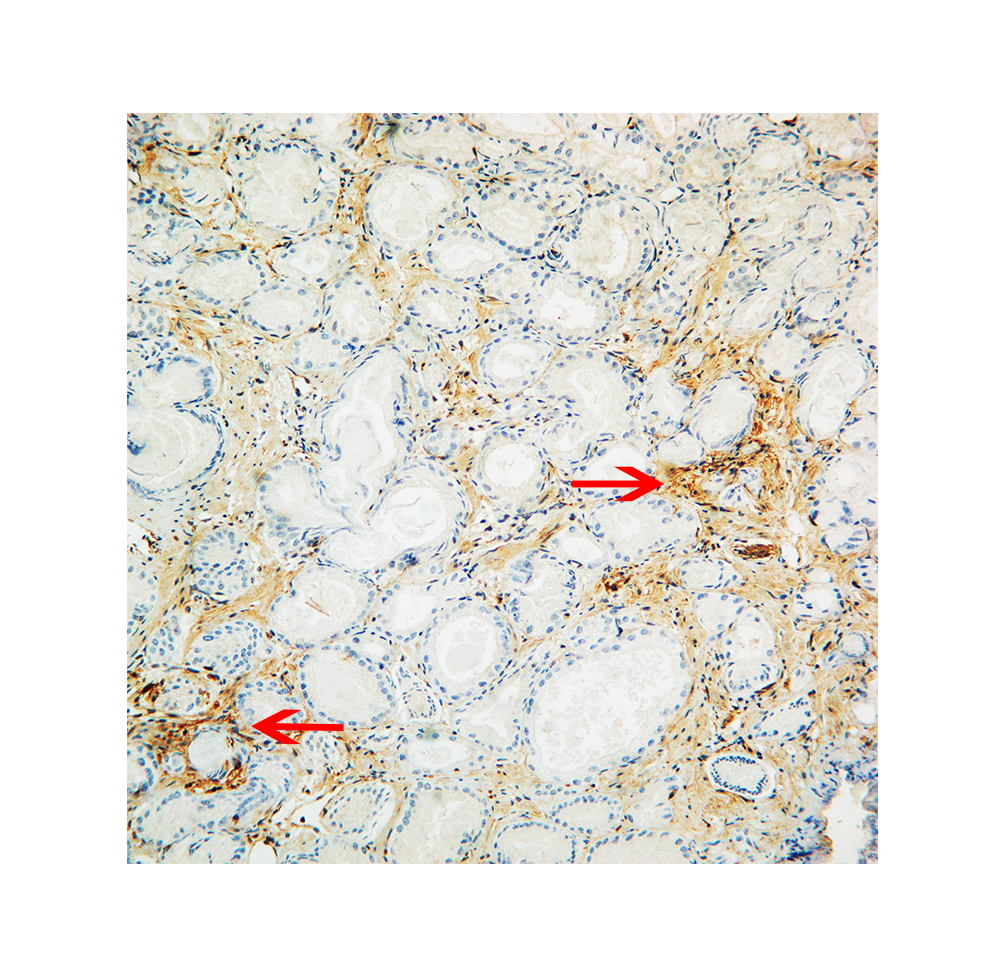

Supplement: Supplemental Figure 2 — Immunohistochemical result of positive GBP1 protein expression in stromal cells of a prostate cancer. Areas of stromal cells (arrows) in a prostate cancer are shown with strong GBP1 protein expression, while the prostate cancer cells are largely negative for GBP1 protein expression (x200). [file Image_2.TIF]
